# Anti-inflammatory substances from onions could be an option for treatment of COVID-19—a hypothesis

**DOI:** 10.1007/s40629-020-00146-2

**Published:** 2020-10-20

**Authors:** W. Dorsch, J. Ring

**Affiliations:** 1 Pediatric Ambulance, Allergology, Pneumology, Natural Methods, Aidenbachstraße 118, 81379 Munich, Germany; 2 Dept Dermatology and Allergology Biederstein, Technical University Munich (TUM), Biedersteiner Straße 29, 80802 Munich, Germany

**Keywords:** Anti-COVID-19 effects (hypothesis), Anti-inflammatory activities, Antithrombotic activities, Antiviral activities, Call for cooperation

Dear editors,

There are three main arguments for onions or onion-based phytopharmaceutical preparations as candidates for the treatment of patients with COVID-19: A broad spectrum of *anti-inflammatory, antithrombotic *and, rather speculative, *antiviral* effects [1, 2].

In 1983, Dorsch and Ring reported to the American Academy of Allergology the inhibitory effects of onions on human allergen-induced late-phase inflammatory reactions [3, 4] and bronchial asthma. Later the active compounds and their mode of action have been identified as inhibitors of leukotriene [5], prostaglandins [5] and thromboxane B biosynthesis in vitro as well as obstructive bronchial reactions in guinea pigs, mice and rats on the inhalation of PAF (platelet activation factor), allergens or sephadex ([6], summary in [7]).

Thiosulfinates, Cepaenes and synthetic analogues (produced by Boehringer-Mannheim) suppress histamine release from human granulocytes and human adenoidal mast cells, leukotriene biosynthesis of human granulocytes (Leukotriene B4 and Leukotriene C4 (LTB4 and LTC4), binding of PAF to human platelets in high concentration, thromboxane biosynthesis of human platelet-rich plasma, lipoxygenase activity of swine leukocytes, cyclooxygenase activity of sheep seminal vesicles, activation of human granulocytes by PAF, chemotaxis of human granulocytes and human lung fibroblasts. Synthetic diphenylthiosulfinate (Boehringer-Mannheim, Germany) hinder in addition proteinase release from human leukocytes, zymosan-induced chemiluminescence of human leukocytes and antigen-related constriction of guinea pig lung parenchymal strips.

In vivo suppression of allergen induced bronchial reactions in guinea pigs, PAF-induced, but not histamine- or acetylcholine-induced bronchial obstruction and bronchial hyperreactivity in guinea pigs has been observed as well as of PAF-induced lethality in mice, multiple bronchospasms in rats and guinea pigs after active and passive immunization and hypersensitization by sephadex and other agents.

**Fig. 1:**
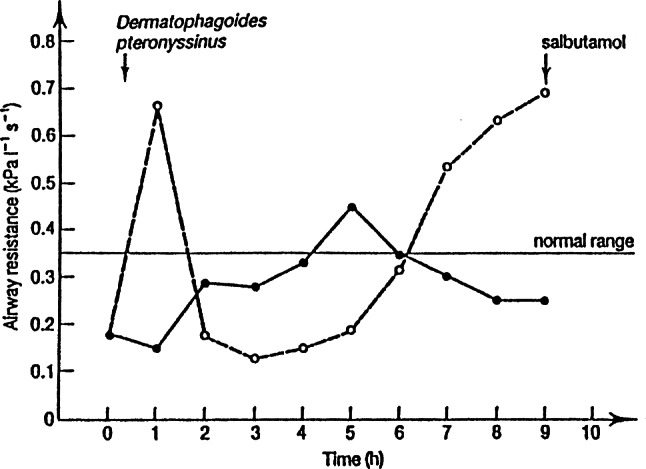
Time course of the bronchial reaction of one adult patient to the inhalation of house dust (dermatophagoides pteronyssinus) after ingestion of 200 ml 5% alcoholic onion extract from 200 g raw onions (*solid line*) or control (*dashed line*)

In humans, local treatment with onion juice can significantly reduce allergic skin inflammation reactions (similar to bee sting reactions) and treat allergic asthma: Fig. 1 shows the inhibitory effect of a 5% alcohol extract from 200 g onions on bronchial obstruction caused by inhalation of house dust mites.

Onions have been used in traditional medicine for thousands of years worldwide. Most of the active substances are not present in intact onions, they will be synthesized de novo in case of injury as defense against predators. The production method is crucial to obtain pharmaceutically active extracts, e.g. heat destroys them. Their pharmaceutic activity corresponds with their instability. But onions remain an important home medicine, they are available worldwide, even in poor countries, they are cheap and could play a role in self-medication in the early stages of COVID-19. In 1999 the WHO assessed the use of the onion as harmless on the whole.

Facing the dramatic development of the pandemic, we report on our scientific efforts at a very early stage hoping for intensive cooperation with other scientists in order to test optimal preparation methods, possible side effects, antiviral and antithrombotic activities in detail. We are currently starting with the following preparation technic even for clinical trials: Peeling 200 g raw yellow onions room temperature, cutting into small cubes, mixing with 80 g dextrose, squeezing, adding alcohol (final 10%), sieving after 2 hours and drinking the daily dose of 100 ml in small portions. Side-effects such as increased bowel activity, flatulence and typical respiratory odour appear tolerable.

Walter Dorsch and Johannes Ring
